# Dietary Patterns and Cardiovascular Disease Risk in Asia

**DOI:** 10.3390/nu15112481

**Published:** 2023-05-26

**Authors:** Hiroyasu Iso

**Affiliations:** Institute for Global Health Policy Research (iGHP), Bureau of International Health Cooperation, National Center for Global Health and Medicine, Tokyo 162-8655, Japan; hiso@it.ncgm.go.jp

The most renowned dietary pattern associated with cardiovascular health is the Mediterranean diet, which is well accepted by Europeans and Americans. However, dietary patterns characterized by high intakes of fish/seafood, soy products, and sodium, and low intakes of meat, dairy products, and calcium are rooted in culture among different races, ethnicities, and countries. Evidence around dietary patterns and cardiovascular health in Asian countries has not been well recognized; however, such evidence has recently emerged. This Special Issue focuses on epidemiological evidence about the association between dietary patterns and cardiovascular disease risk through cross-sectional and prospective cohort studies in Asia. Authors working on established epidemiological studies in each of these countries are invited to submit original or review articles. We have a paper on the cross-sectional study of carotid atherosclerosis [1]; six papers on prospective cohort studies on metabolic risk factors [2,3,4], cardiovascular [5], and related chronic diseases [6,7]; and two review papers on cardiovascular health [8] and wellbeing/longevity [9].

A cross-sectional analysis of 1803 Japanese men and women aged 30–84 years showed that omega-3 fatty acid intake mainly from fish and seafood was inversely associated with a lower prevalence of elevated carotid intima media thickness, an established marker of carotid atherosclerosis [1]. According to a prospective cohort study of 2944 Japanese government employees, a dietary pattern characterized by high intakes of soy, fruit, vegetables, and seaweed was inversely associated with the risk of metabolic syndrome, while another dietary pattern characterized by milk/dairy products, meat, rice, and pickles was positively associated with the risk [2]. A typical Japanese dietary pattern, characterized by high intakes of beans, fish, fruit and vegetables, soy products, and rice was inversely associated with the risk of diabetes mellitus from a prospective cohort study of 22,740 Japanese men and women aged 20–89 years [4]. A rice-based diet characterized by higher intakes of soy products and seaweed and lower intakes of meat and eggs was inversely associated with the risk of cardiovascular disease mortality in men but not in women according to a Japanese cohort study of 13,355 men and 15,724 women aged 35 years or more [5]. A cohort analysis of 42,126 Chinese men and women aged 30–79 years from the China Kadoorie Biobank showed that a dietary pattern characterized by high intakes of fresh fruit and protein-rich foods, such as soybean, meat, poultry, fish and eggs, and dairy products, was inversely associated with the risk of chronic obstructive pulmonary disease [6]. A Japanese diet index, allocating one point for the sex-specific median-or-more intakes of rice, miso soup, seaweeds, fish, green and yellow vegetables, and green tea, and for less than the sex-specific median intakes of beef, pork, and coffee, totaling 0 to 9 pints, was inversely associated with the risk of dementia [7]. The Healthy Taiwanese Eating Approach toward total well-being and health longevity was proposed by reviewing 17 cross-sectional and 7 cohort Taiwanese studies, covering a wide range of health conditions, such as allergic diseases and various cardio-metabolic diseases, as well as cancer, frailty, cognitive decline, and all-cause mortality [9]. That approach featured higher intakes of plant-based foods and drinks (tea), aquatic foods, and soy products but lower intakes of fast foods, fatty and processed meats, sugar, and salt-rich foods/drinks. This dietary pattern is consistent with the Mediterranean diet but with unique culture-specific features, such as more soy products and tea but less fried rice or noodle products, and so on.

The above articles addressed the beneficial effects of dietary patterns in Asia on various health outcomes, including cardiovascular disease; however, the following articles studied their potential adverse effects.

In a cohort analysis of 17,318 Chinese men and women from the China Kadoorie Biobank, they derived two lipid-metabolism-related dietary patterns. One of the patterns, characterized by high intakes of fish, poultry, and other staples, as well as fresh fruit and vegetables, was correlated with a higher body mass index, waist circumference, and LDL cholesterol, and positively associated with the risk of diabetes mellitus [3]. The impact of dietary fructose and a high salt diet on hypertension and cardiovascular risk was discussed through selected studies in Japan, China, and South Korea [8]. They reviewed dietary sodium consumption through 12 studies and dietary fructose intake from 8 studies, indicating that people in these countries tended to have lower fructose and higher salt intake compared with Western populations. Fructose, derived mainly from sugar-sweetened beverages, enhances intestinal sodium absorption and renal tubule sodium absorption with sustained levels of plasma renin activity, and thus fructose and salt intake could have an addictive effect on increasing blood pressure levels. An increase in fructose intake among adolescence, as seen in some Asian countries, if high sodium intake is sustained, may threaten an increased risk of hypertension in the future.

The studies featured in this Special Issue provided evidence of the beneficial effects of dietary patterns observed commonly in Japan, China, and South Korea on cardiovascular disease and other related health conditions. These dietary patterns have similar features to the Mediterranean diet but with more soy products, tea, rice, seaweeds, and so on. However, some of the dietary patterns could have potential adverse effects on metabolic risk factors and hypertension when people have increased intakes of sugar-sweetened beverages and energy and a decrease in physical activity.

Policymakers who wish to enhance primary care through dietary intervention need to understand the beneficial and adverse effects of dietary patterns in their own countries and make healthy-diet policies. Primary-care professionals and providers must educate high-risk individuals and patients to encourage the adoption of healthy dietary patterns to minimize adverse features and prevent cardiovascular disease, the No. 1 cause of mortality worldwide.
